# Regulation of Synaptic Vesicle Docking by Different Classes of Macromolecules in Active Zone Material

**DOI:** 10.1371/journal.pone.0033333

**Published:** 2012-03-16

**Authors:** Joseph A. Szule, Mark L. Harlow, Jae Hoon Jung, Francisco F. De-Miguel, Robert M. Marshall, Uel J. McMahan

**Affiliations:** 1 Department of Neurobiology, Stanford University School of Medicine, Stanford, California, United States of America; 2 Department of Physics, Stanford University School of Humanities and Sciences, Stanford, California, United States of America; 3 Department of Biology, Texas A&M University, College Station, Texas, United States of America; 4 Instituto de Fisiología Celular-Neurociencias, Universidad Nacional Autónoma de México, Distrito Federal, Mexico; Claremont Colleges, United States of America

## Abstract

The docking of synaptic vesicles at active zones on the presynaptic plasma membrane of axon terminals is essential for their fusion with the membrane and exocytosis of their neurotransmitter to mediate synaptic impulse transmission. Dense networks of macromolecules, called active zone material, (AZM) are attached to the presynaptic membrane next to docked vesicles. Electron tomography has shown that some AZM macromolecules are connected to docked vesicles, leading to the suggestion that AZM is somehow involved in the docking process. We used electron tomography on the simply arranged active zones at frog neuromuscular junctions to characterize the connections of AZM to docked synaptic vesicles and to search for the establishment of such connections during vesicle docking. We show that each docked vesicle is connected to 10–15 AZM macromolecules, which fall into four classes based on several criteria including their position relative to the presynaptic membrane. In activated axon terminals fixed during replacement of docked vesicles by previously undocked vesicles, undocked vesicles near vacated docking sites on the presynaptic membrane have connections to the same classes of AZM macromolecules that are connected to docked vesicles in resting terminals. The number of classes and the total number of macromolecules to which the undocked vesicles are connected are inversely proportional to the vesicles’ distance from the presynaptic membrane. We conclude that vesicle movement toward and maintenance at docking sites on the presynaptic membrane are directed by an orderly succession of stable interactions between the vesicles and distinct classes of AZM macromolecules positioned at different distances from the membrane. Establishing the number, arrangement and sequence of association of AZM macromolecules involved in vesicle docking provides an anatomical basis for testing and extending concepts of docking mechanisms provided by biochemistry.

## Introduction

The cytoplasm of axon terminals at the nervous system’s chemical synapses includes membrane-bound synaptic vesicles, which contain neurotransmitter molecules. Some vesicles are docked on (held in close association with) the presynaptic plasma membrane in specialized regions called active zones [1], [2]. When an impulse arrives at an axon terminal, some of the docked vesicles fuse with the presynaptic membrane to form a pore through which the vesicles’ neurotransmitter is secreted into the synaptic cleft to generate a response in the terminal’s target cell [3], [4]. Vesicles that have fused with the presynaptic membrane are replaced at the docking sites in active zones by previously undocked vesicles [5]. Both the docking of vesicles on the presynaptic membrane and their fusion with it are mediated by the interaction of proteins, some of which are linked directly to the vesicle and presynaptic membrane. Such proteins have been characterized biochemically, and much is known about the nature of the interactions that lead to docking and fusion [6].

Other major components of active zones are aggregates of macromolecules in the presynaptic membrane and dense aggregates of macromolecules called active zone material (AZM), which are attached to the membrane and extend several tens of nanometers into the cytoplasm [1], [7]. The aggregates of macromolecules in the presynaptic membrane include voltage-gated calcium channels [8]–[10]. It is well-established that an impulse in the axon terminal causes the calcium channels to open, and the influx of calcium into the cytoplasm triggers the protein mediated fusion of docked vesicles with the presynaptic membrane [3], [6]. The prominence of AZM at active zones and its close proximity to docked vesicles and calcium channels make it seem likely that AZM plays a role in the docking of synaptic vesicles on the presynaptic membrane and/or the fusion of the vesicles with the membrane [11]. However, the small size, high density and complex arrangement of macromolecules in the AZM have made it difficult to correlate AZM structure with active zone function.

One way of exposing the architecture of the AZM for studying its association with other components of the active zone is by applying electron tomography to tissue sections from synapses. Electron tomography uses a series of two-dimensional (2-D) transmission electron microscope images from a specimen taken at different tilt angles to generate a three-dimensional (3-D) reconstruction of the specimen [12]. The AZM macromolecules are much narrower than the thinnest tissue sections that can be cut (∼30 nm), and they overlap each other in a section’s depth axis. However, they can be seen distinctly in serial virtual slices made through the reconstructed volume that are thinner than the macromolecules. The macromolecules can, then, be studied in 3-D either alone or together with other structures by using the serial slices for segmenting them from the volume and generating surface models of them (e.g. [11], [13], [14]).

The AZM at active zones of neuromuscular junctions (NMJ’s) of the frog is particularly convenient for electron tomography studies. Its overall shape is relatively simple, as are the arrangement of its associated docked vesicles and calcium channels [1], [8]–[10]. It is also well established that, at the frog’s NMJ, the membrane of former docked vesicles that have fused with the presynaptic membrane moves laterally within the presynaptic membrane to reform vesicles at sites away from the active zone [15]–[17]. Previous electron tomography studies on the active zones at resting frog NMJ’s fixed with glutaraldehyde and stained with heavy metals have shown that the AZM is composed of a network of elongate macromolecules, and that individual docked vesicles are connected to one end of many of them. The connection sites of the macromolecules on each vesicle are broadly distributed over the vesicle hemisphere that faces the AZM. A detailed analysis of the AZM network within 15 nm from the presynaptic membrane showed that it is composed of three logically distinct classes of macromolecules called beams, ribs and pegs [11], [13]. Beams are connected to beams and ribs, ribs are also connected to docked vesicles and pegs, and pegs, based on the frequency and distribution of their connections to the presynaptic membrane, are thought to be connected to the macromolecules in the membrane that include calcium channels [11]. Beams, ribs and pegs, similarly linked to docked vesicles and calcium channels, have also been identified in the AZM of mouse NMJ’s [14]. Such findings have led to the conclusion that the AZM is a multifunctional organelle that helps dock synaptic vesicles on the presynaptic membrane and anchor calcium channels in the membrane. It may also play a role in the fusion of docked vesicles with the membrane. By extension, many, if not all, of the proteins thought from biochemical experiments to mediate the vesicles’ docking on and fusion with the presynaptic membrane may well contribute to the composition of AZM macromolecules.

The AZM at the frog’s NMJ projects several 10’s of nm from the presynaptic membrane into the cytoplasm. Thus, the beams, ribs and pegs account for only a small fraction of the AZM’s macromolecules. Moreover, the ribs comprise only a small fraction of the AZM macromolecules connected to each docked vesicle [11]. Here, we defined the arrangement and associations of macromolecules throughout the AZM in heavy metal stained terminals fixed at rest, with a view to characterizing all AZM macromolecules connected to docked vesicles. We, then, determined in stained terminals, fixed during evoked synaptic activity, whether the same macromolecules connected to docked vesicles in resting terminals are connected to undocked vesicles near docking sites on the presynaptic membrane vacated by those docked vesicles that had fused with the membrane during synaptic transmission. The results provide evidence that several classes of AZM macromolecules direct undocked vesicles to docking sites on presynaptic membrane by an orderly series of interactions, and that the same classes of macromolecules help maintain the vesicles at the docking sites until the vesicles fuse with the membrane during synaptic transmission.

## Results

### Active Zone Overview

**Figure 1:**
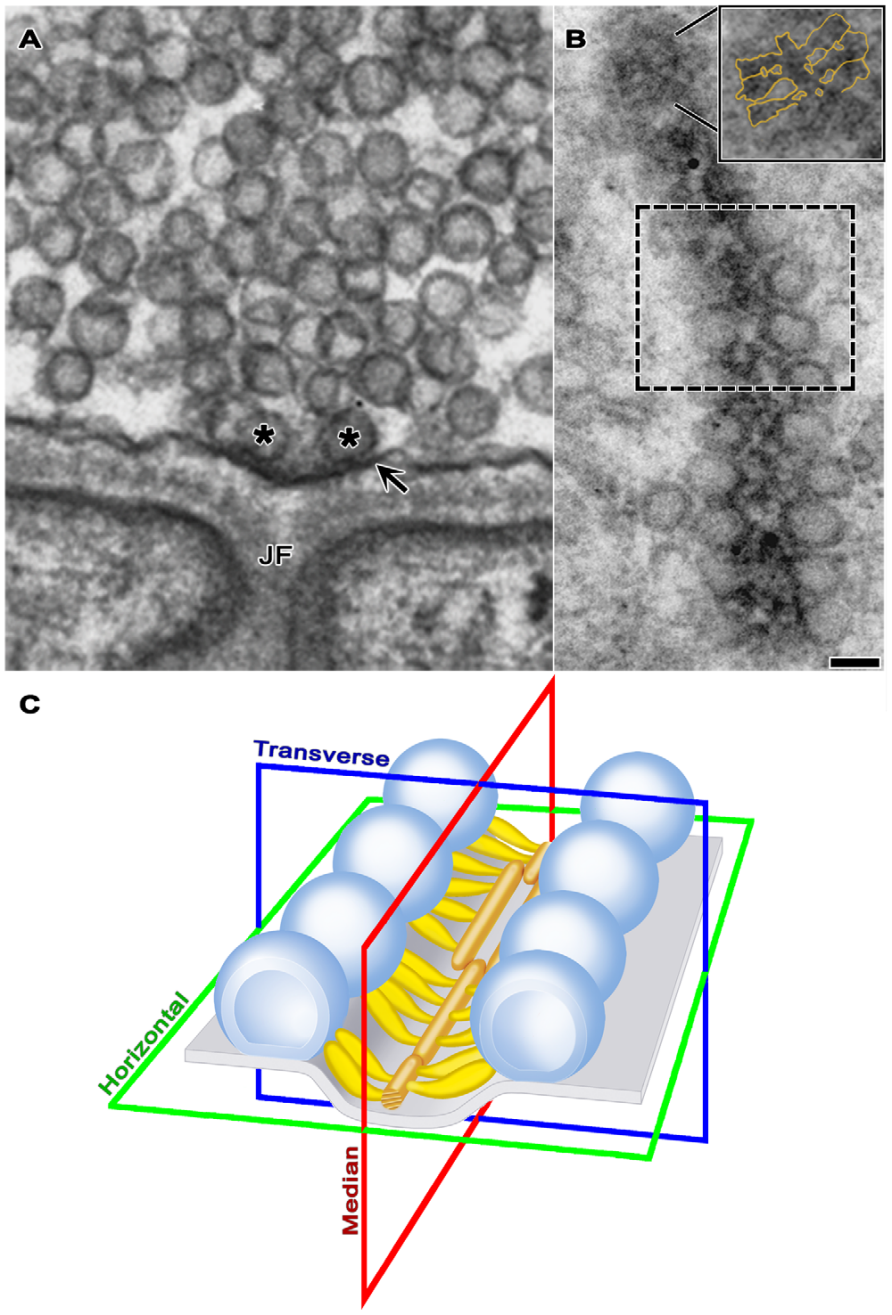
Layout of the active zone. **A**) Viewed in the active zone’s transverse plane by conventional 2-D electron microscopy of a tissue section, the AZM is a small electron dense patch between a pair of synaptic vesicles (asterisks) docked on the presynaptic membrane (arrow). The AZM’s superficial surface is attached to the presynaptic membrane where the membrane curves into the synaptic cleft forming the active zone ridge just opposite the mouth of a junctional fold (JF) in the muscle fiber’s surface. The AZM’s irregular deep surface extends about 75 nm into the cytoplasm. A cloud of undocked vesicles lies lateral and deep to the docked vesicles. **B**) Viewed in the active zone’s horizontal plane by conventional 2-D electron microscopy of a tissue section, the AZM is a narrow, irregularly dense band. This band of AZM has a slight angular change midway along its length (dashed box). The section includes a row of docked synaptic vesicles on each side of the AZM band in the lower half of the image. It passed superficial to the rows of docked vesicles along much of the band in the upper half of the image, where it includes only that portion of the band in the active zone ridge. At the tip of the AZM band in the upper half of the image, the section includes only the superficial layer of the AZM, exposing a series of ribs extending from each side of a beam, which are outlined in gold in the inset. Scale bar in **A** and **B** = 50 nm. **C**) 3-D schematic of the active zone, showing the active zone ridge in the presynaptic membrane (pale blue), rows of docked synaptic vesicles (dark blue), the AZM’s ribs (yellow gold), beams (brown gold) (from electron tomography on tissue sections; [11], [13]), and indicators of the active zone’s horizontal, transverse and median planes.

Active zones at frog NMJ’s are arranged in narrow bands on the presynaptic membrane (Figure 1; see also [1], [7], [11]). The bands can be more than a micrometer long, and they run orthogonal to the long axis of the axon terminal, with occasional, slight angular changes along their length. The main body of AZM extends throughout the length of each active zone. Its depth varies at regular intervals along its length. Its deepest points extend ∼75 nm from the presynaptic membrane into the cytoplasm. Its width is ∼50 nm. A row of docked vesicles (50–60 nm in diameter [14]) lines each side of the main body of AZM. Numerous undocked vesicles of similar size are situated lateral and deep to the active zone. At the 2–3 nm spatial resolution provided by our tomography methods, the membrane of docked vesicles directly contacts the presynaptic membrane; no gap is seen between the membranes, and the average distance from the luminal surface of the vesicle membrane to the extracellular surface of the presynaptic membrane in the region of contact is twice the average distance across each of the two membranes beyond the region of contact (Jung and McMahan, unpublished results). The portion of the presynaptic membrane next to the AZM curves into the synaptic cleft (Figure 1A and C) to form the active zone ridge. The ridge is situated just opposite an infolding (junctional fold) in the surface of the muscle fiber. Along each slope of the ridge, the membrane contains a linear array of macromolecules paralleling the long axis of the AZM and the rows of docked vesicles (see below). The membrane macromolecules are thought to include voltage gated calcium channels and calcium activated potassium channels [8]–[10].

**Figure 2:**
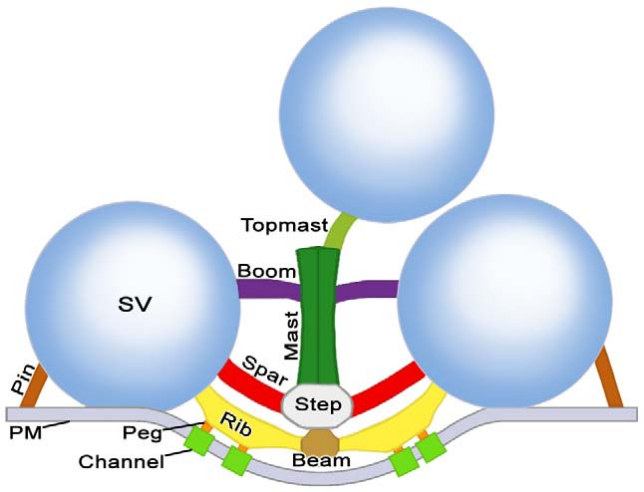
Composite diagram of layers of AZM macromolecules at resting active zones exposed by electron tomography. Shown in the transverse plane of the active zone, the superficial layer of macromolecules in the main body of the AZM includes beams, ribs and pegs; the intermediate layer includes steps and spars; and the deep layer includes masts, booms and topmasts. Ribs, spars and booms connect to docked synaptic vesicles (SV), topmasts connect to nearby undocked vesicles, and pegs connect to macromolecules in the presynaptic membrane (PM). Pins are positioned away from the main body of the AZM and link docked vesicles to the presynaptic membrane. All AZM components are shown in virtual slices and surface models from reconstructed tissue sections in subsequent Figures except for pegs, which were not included in this study (but see [11], [13]). The color code is the same for all Figures.

We distinguished by electron tomography three contiguous layers of logically distinct classes of macromolecules in the main body of AZM (Figure 2). Each layer is situated at a different depth from the presynaptic membrane. The superficial layer lies adjacent to the presynaptic membrane, is ∼15 nm thick and contains the previously described beams, ribs and pegs [11]. The intermediate layer is ∼15 nm thick and contains two classes of macromolecules termed steps and spars. The deepest layer varies in thickness up to ∼45 nm and includes three classes of macromolecules named masts, booms and topmasts. Here we characterize the classes of macromolecules in the intermediate and deep layers and relate them to the macromolecules in the superficial layer and to the connections of AZM macromolecules on docked and nearby undocked vesicles. We also describe a class of AZM macromolecules, called pins, which are separate from the main body of AZM. Pins directly link docked vesicles to the presynaptic membrane.

As a result of electron tomography studies demonstrating the simply arranged beams and ribs in the superficial layer, these macromolecules can now be recognized in favorable tissue sections imaged by conventional 2-D electron microscopy (see inset in Figure 1B). The same images show that the AZM deep to the superficial layer has a varied appearance along the length of the AZM band, but, thus far, we have been able to identify distinct macromolecules in these layers only by tomographic analysis.

**Figure 3:**
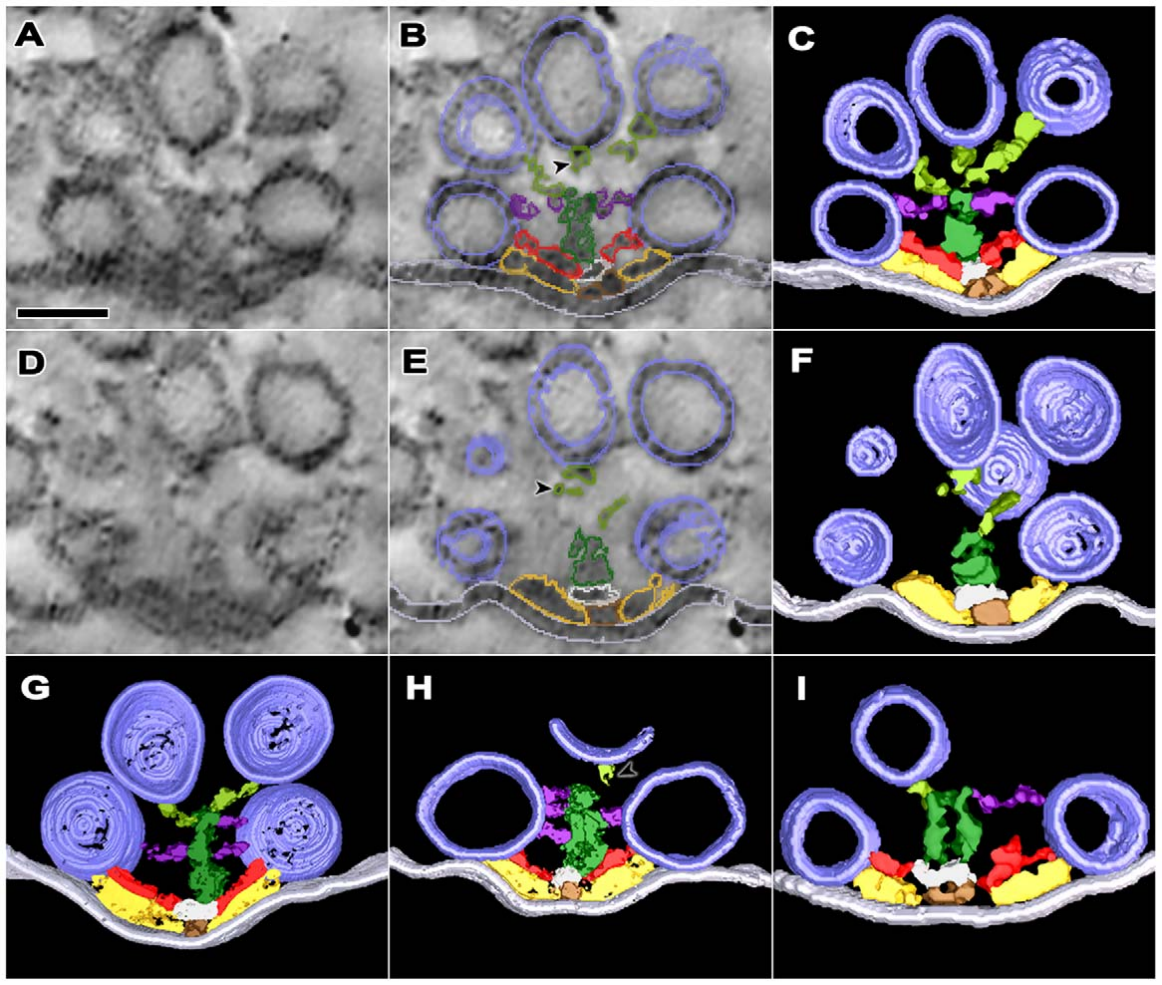
Transverse distribution of macromolecules in the main body of the AZM at resting active zones. **A,D)** Two regions 10 nm apart along the depth axis of the reconstructed volume of an active zone sectioned near its transverse plane. Each of the images was formed by the summation of three 1.2 nm thick serial virtual slices made through the volume in the same plane as the section. **B,E)** The same regions shown in **A** and **D** with the summed outlines of the AZM macromolecules in each of the virtual slices overlaid. **C,F)** Surface models, 10 nm thick, of the AZM macromolecules shown in **A** and **D** derived from segmentation of eight adjacent virtual slices and rotated to the transverse plane. **G,H,I)** Surface models generated as for those in **C** and **F**. Tissue used for each of the surface models was from a different frog. The tissue was chemically fixed except for that used for the surface model in **I,** which was fixed by rapid freezing. Ribs (yellow gold) extend from beams (brown gold) in the superficial layer of AZM, spars (red) extend from steps (gray) in the intermediate layer, and booms (purple) extend from masts (dark green) in the deep layer, while the ribs, spars and booms connect to synaptic vesicles (dark blue) docked on the presynaptic membrane (pale blue). Topmasts (light green) in **C,F,G** and **I** link masts to undocked vesicles near the active zone’s midline. The linkage of some topmasts to masts is incomplete in **C,F,** and **H** because discontinuous staining made segmentation uncertain or the topmast extended beyond the edge of the tissue section (arrowheads). Scale bars  = 50 nm.

In the following account, the horizontal plane (Figure 1C) of an active zone is parallel to the presynaptic membrane beyond the active zone ridge. A median plane is orthogonal to the horizontal plane and parallel to the long axis of the AZM. A transverse plane is orthogonal to both the horizontal plane and the median plane. For convenience, much of our analysis was done on sections from muscles fixed and stained by bathing them in glutaraldehyde and, subsequently, in osmium tetroxide. However, as indicated in Figure 3, all structures and relationships observed in aldehyde fixed tissue were also seen in tissue fixed by rapid freezing and initially stained by freeze-substituting osmium tetroxide, which can be superior for the preservation of certain structures [18]. Regardless of the method of preparation, we observed by electron tomography no significant difference in the presence and relationships of AZM macromolecules, in the diameter of synaptic vesicles and in the relationship of docked vesicles to the presynaptic membrane. The resting active zones used for data in this study were derived from 5 muscles. In no case did we observe vesicles fused with the presynaptic membrane at active zones in these preparations.

### Main Body of the AZM at Resting Axon Terminals

**Figure 4:**
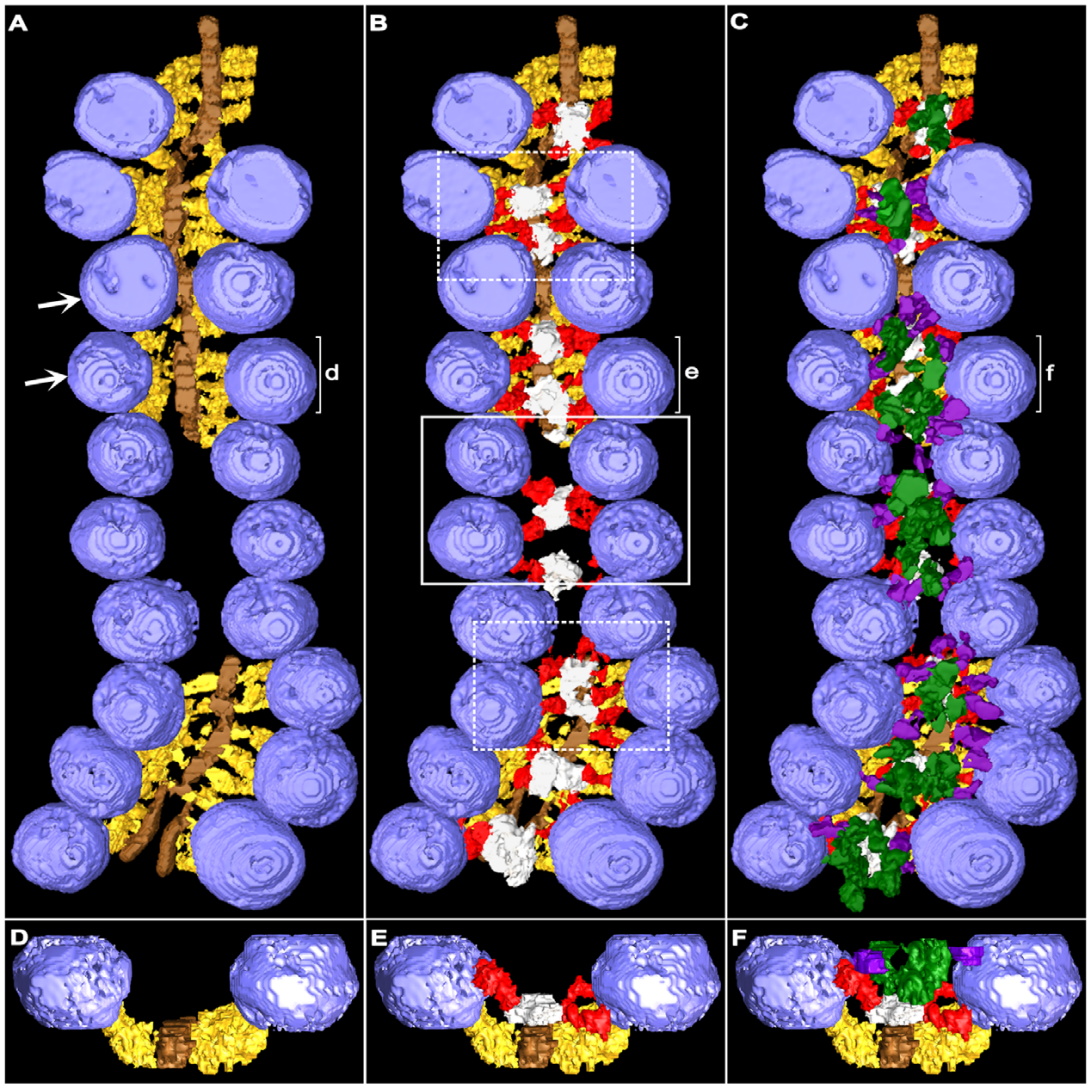
Longitudinal distribution of the AZM’s main body macromolecules at resting active zones. Surface models were generated from 1.0 nm thick serial virtual slices through a reconstructed active zone sectioned near its horizontal plane. The slice series was in the same plane. **A**) The ribs (yellow gold) and beams (brown gold) of the superficial layer of the AZM extend throughout the length of the active zone except at the gap, where the superficial layer was not included in the tissue section. Multiple ribs connect to each docked vesicle except at the upper right, where the vesicle(s) was too close to the edge of the section to be clearly discerned in the reconstruction. Ribs in some regions are not distinguishable from their neighbors because of the model’s angle of rotation. **B**) The intermediate layer shown together with the superficial layer. Steps (gray) are centered between opposing pairs of docked vesicles (as in solid box) along straight stretches of the active zone. The positioning of the steps is less regular where there is an angular change in the active zone’s long axis (dashed boxes). Typically, each docked vesicle is connected to spars (red) arising from two steps. **C**) The deep layer is shown together with the superficial and intermediate layers. Masts (dark green) overlay steps (compare with **B**). Multiple booms (purple) extend from each mast to connect to docked vesicles. **D,E,F**) Near transverse views of the surface models shown in **A**,**B**, and **C** from the regions in those panels marked **d**, **e** and **f** respectively. Arrows in **A** indicate the vesicles and associated AZM macromolecules used in Figure 5 to demonstrate our method for measuring the angle of approach of different classes of AZM macromolecules to docked vesicles. Topmasts were not included in the tissue section.

Steps were connected to beams (Figures 2,3,4). Their long axis ran parallel to the median plane of the AZM, as did the long axis of beams. However, they were ∼28 nm long compared to the average length of beams, which was ∼75 nm (Table 1). In cross section, the diameter of the steps in the horizontal plane was ∼22 nm, which was greater than the ∼11 nm average diameter of the beams. The diameter of the steps vertical to the presynaptic membrane was ∼14 nm (Table 1).

**Table 1:** Averaged Dimensions of AZM Macromolecules in Resting Active Zones.

AZM Macromolecule	Length±SD (nm)	N	Diameter±SD (nm)	N	# of Data Sets
Beams	75.1±13.1	6	10.7±0.7	12	3
Steps	28.4±7.6	14	21.8±5.8 (Horiz.)	16	4
			13.9±2.7 (Vert.)	14	
Masts	31.5±4.5	6	22±3	6	5
Ribs	27.9±7.7	53	9.4±1.4	88	6
Pins	8.6±3.5	32	5.0±1.0	17	4
Spars	17.6±6.5	56	7.1±1.2	58	5
Booms	16.0±4.8	60	6.5±1.3	67	6
Topmasts	24.6±9.4	6	6.9±2.1	6	3

While beams were distributed continuously along the length of an active zone, the steps were positioned at intervals. For the active zone shown in Figure 4, the center-to-center spacing of the steps was ∼50 nm (48.8±14.9 nm SD). Along straight regions of the active zone, the steps were roughly centered between a tetrad of docked vesicles: two adjacent vesicles in one row and two adjacent vesicles in the opposite row (see solid white box in Figure 4B). The alignment of vesicles with steps was less regular, however, where there was an angular change along the course of the AZM (see dashed boxes in Figure 4B).

Spars were filamentous and extended from the steps to the membrane of docked vesicles (Figures 2,3,4). Where steps were positioned between a tetrad of vesicles along straight stretches of the active zone, typically four spars radiated from each step (4.2±1.1 SD; n = 13 steps), and each of the spars connected to one of the vesicles of the tetrad. Accordingly, each vesicle in a tetrad was usually connected to two spars (2.2±0.5 SD; n = 20 vesicles), which originated from two different steps (Figure 4B). Where the active zone curved, the steps were sometimes larger than along straight stretches. The number of spars radiating from such steps was greater than the number for smaller steps (see the dashed boxes in Figure 4B). Nevertheless, all of the spars arising from the steps in curved regions of the active zone contacted nearby docked vesicles, as did all those arising from steps in the straight region.

**Figure 5:**
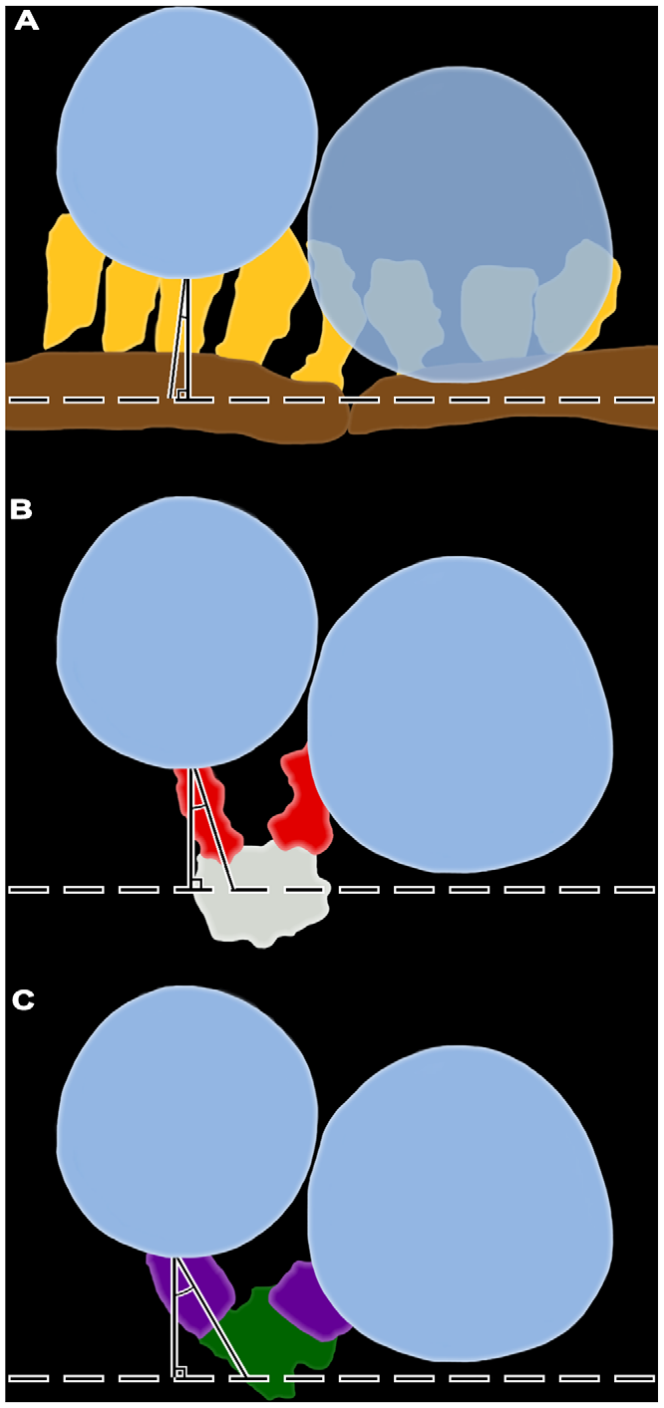
Angle of approach of main body AZM macromolecules to docked vesicles. The docked vesicles shown at the arrows in Figure 4 together with selected members of their associated AZM macromolecules were projected and traced onto a two dimensional plane. Lines drawn parallel to the ribs (yellow gold; **A**), spars (red; **B**) and booms (purple; **C**) approach a vesicle at different angles relative to perpendicular lines drawn to the plane of the beam (brown gold). Measurements of such angles from many docked vesicles reveal that the average angle of approach is significantly different for each class of AZM macromolecule, as detailed in the text and as shown here for single macromolecules from each class: **A**, 5°; **B**, 17°; and **C**, 27°.

**Figure 6:**
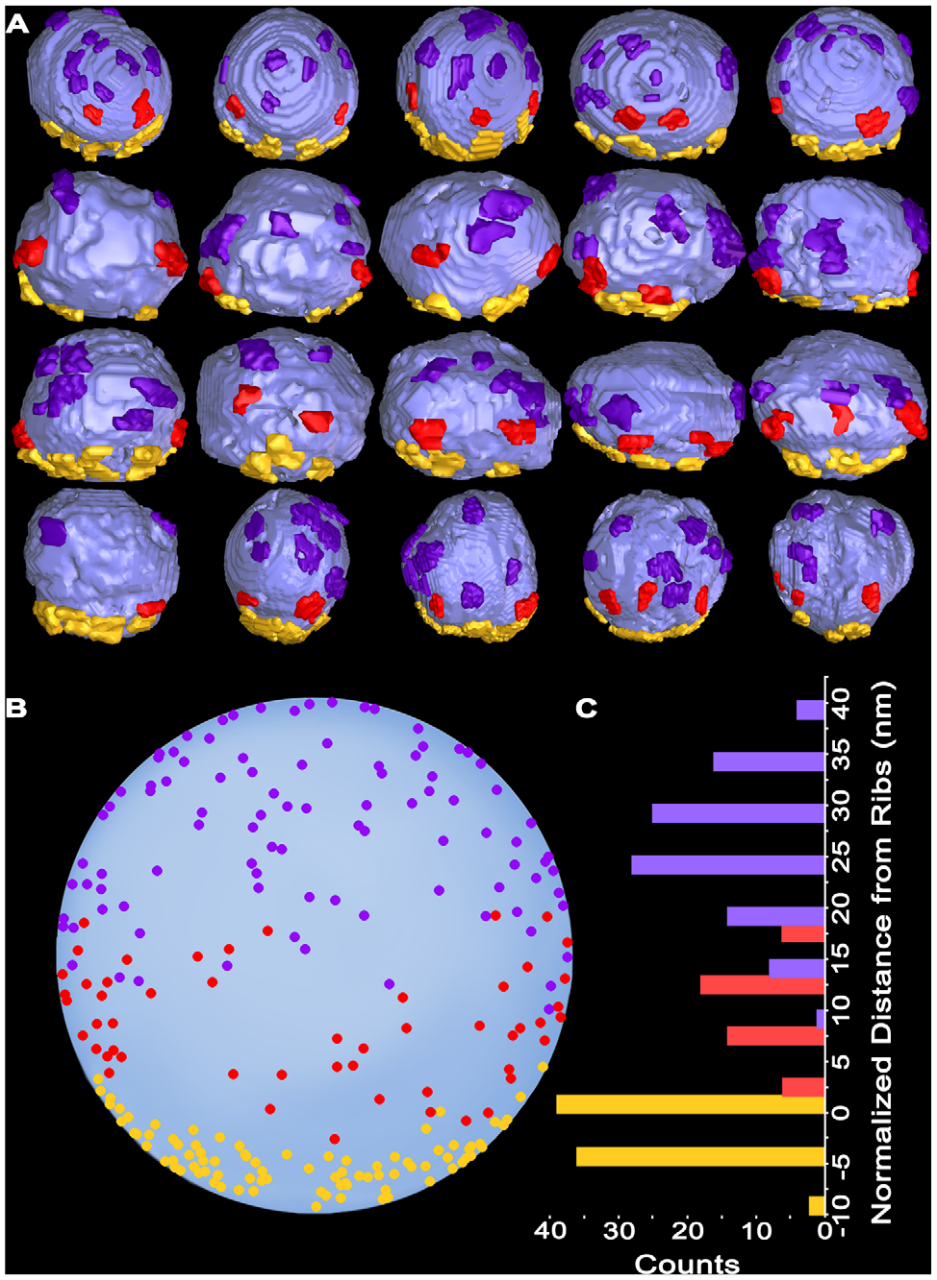
Connection sites of main body AZM macromolecules on docked vesicles at resting active zones. **A)** Median view of 20 docked vesicles with ∼3 nm long stretches of ribs (yellow gold), spars (red) and booms (purple) attached to mark their connection sites on the vesicles. **B)** The centroids of AZM connection sites on all vesicles shown in **A** were normalized for small variations in vesicle diameter. They were, then, aligned to the relative position of the rib connections using a cross-correlation algorithm, and plotted on an idealized sphere. **C)** The rib, spar and boom connection sites on the idealized sphere in **B** plotted according to their distance from the rib connections. There is little overlap between vesicle domains connected to ribs and spars and moderate overlap between domains connected to spars and booms, but the domains are distinct from each other with a significance level of p<0.0001.

Spars ran nearly parallel to the presynaptic membrane just deep to the ribs (Figures 2,3,4). They were ∼18 nm long, and their average diameter was ∼7 nm. These dimensions were less than the average dimensions of ribs (p<0.0001, t-test), which were ∼28 nm long and ∼9 nm in diameter (Table 1). The spars were separated from the ribs by a gap up to 5 nm wide. The spars also typically approached the docked vesicles to which they were connected at a different angle than the ribs (Figure 5A,B). For spars, the average angle of approach was ∼23° (23.3°±13.1° SD; n = 44 spars), which is significantly different from the ∼11° (10.6°±6.2° SD; n = 32 ribs) angle that ribs approached the vesicles (p<0.0001, t-test). While each docked vesicle was connected to 2 spars, it was in most cases connected to 4 ribs (3.9±0.6 SD, n = 20 from 3 data sets). Thus, all ribs were not apposed by spars (Figure 4B,6A).

Masts were connected to the deep surface of each step (Figures 2,3,4). They extended ∼32 nm from the step, vertical to the presynaptic membrane. Their horizontal profile near the mast-step boundary was nearly circular (∼22 nm diameter; Table 1), which was characteristically different from the elongate horizontal profile of steps. While steps stained nearly uniformly, the staining of masts was distinctively discontinuous. In cases where the staining and slice plane were favorable, masts were seen to be composed of 4–9 serpentine strands that extended along the mast’s long axis (Figure 3I). The average diameter of particularly well-stained strands was 9±2 nm SD (n = 5 masts from 4 data sets).

The filamentous booms arose from the masts ∼39 nm (39.0±7.7 nm SD; n = 26 booms from 5 data sets) from the presynaptic membrane and ∼12 nm (11.6±10.3 nm SD; n = 22 booms from 4 data sets) from the deep end of the masts (Figures 2,3,4). They were ∼16 nm long and had an average diameter of ∼7 nm (Table 1). They extended from the masts to terminate on docked vesicles. Along straight stretches of the active zone, where the masts and their steps were positioned between tetrads of docked vesicles, 8–14 booms (10.6±1.8 SD; n = 8) radiated from a mast to terminate on each of the vesicles of a tetrad in nearly equal numbers. Thus, each docked vesicle was, on average, connected to 5.0±2.0 SD booms from two masts (Figures 4C,6A). Each boom approached the vesicle at an average angle of ∼30° (30.0°±14.7° SD; n = 40 booms) (Figure 5C), which was significantly greater than the angles that spars and ribs approached the vesicle membrane, as described above (p<0.01; determined by ‘Analysis of Variance’-ANOVA).

Topmasts were elongate macromolecules that linked the deep end of the masts to the membrane of undocked vesicles next and deep to the docked vesicles (Figures 2,3). On average 1–2 topmasts arose from a mast, and at least one undocked vesicle was connected to masts through a topmast with an 88% occurrence (15 of 17 masts from 7 data sets). Topmasts were oriented at various angles relative to the long axis of the masts. For 6 topmasts in 3 data sets the average length was ∼25 nm and average diameter was ∼7 nm (Table 1). We did not seek to determine the relationship of topmasts and booms to the strands that compose the masts, all of which had a similar diameter.

### Pins at Resting Axon Terminals

**Figure 7:**
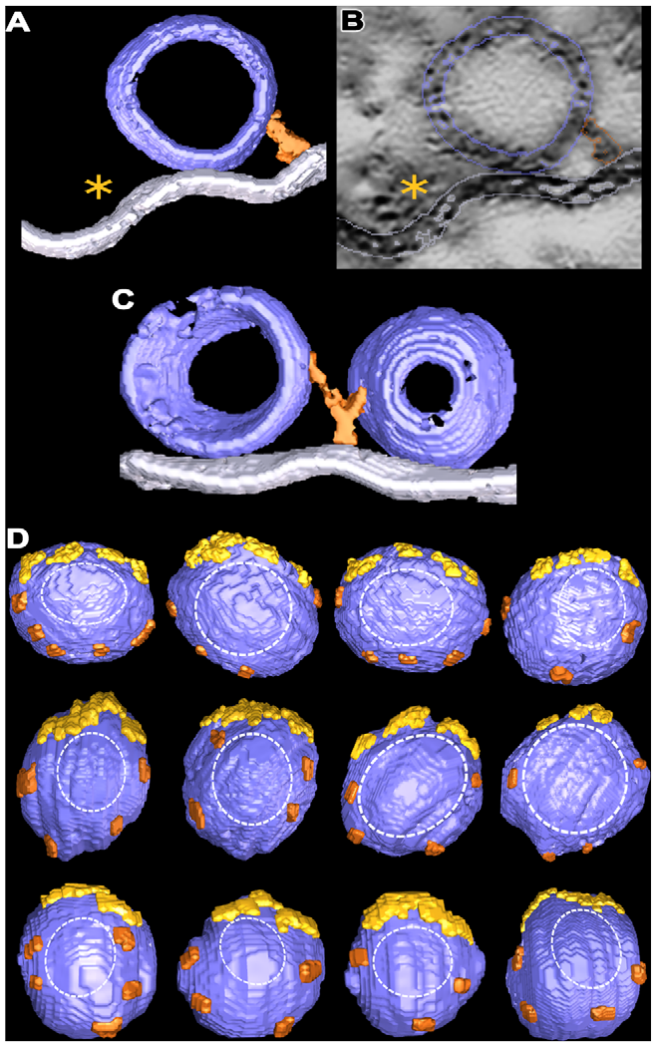
Arrangement of pins at resting active zones. A) A surface model, 9 nm thick, from an active zone sectioned in the transverse plane. A pin (copper) links the hemisphere of a docked vesicle that faces away from the active zone ridge (asterisk) to the presynaptic membrane. **B)** A virtual slice, 0.5 nm thick, from the series of virtual slices segmented to generate the surface model in **A.** The pin, docked vesicle and presynaptic membrane are outlined. An asterisk marks the main body of the AZM in the active zone ridge. **C)** A surface model, 20 nm thick, from an active zone sectioned in the median plane. The docked vesicles are from a row of such vesicles flanking the main body of AZM. A pin between the vesicles bifurcates to link the vesicles to the presynaptic membrane. **D)** The connection sites of pins and ribs (marked by ∼3 nm long segments of each: ribs, yellow gold; pins, copper) on docked synaptic vesicles viewed in the horizontal plane. The vesicles are from four active zones that were sectioned in the median or transverse planes after fixation by glutaraldehyde or rapid freezing. The connection sites surround the region of contact between the vesicle membrane and presynaptic membrane (dashed outline).

Pins arose from the presynaptic membrane lateral to the main body of the AZM and the active zone ridge to terminate on docked vesicles (Figures 2,7). On average, ∼4 pins (4.1±0.7 SD; a range 3 to 5) were connected to each docked vesicle. The average length of the pins was ∼9 nm (Table 1). Typically, a pin terminated on only one vesicle, but some pins between docked vesicles bifurcated to terminate on each (Figure 7C).

### Distribution of the Connection Sites of AZM Macromolecules on Docked Vesicles in Resting Axon Terminals

The arrangement of ribs, spars and booms in distinct layers of the AZM, each situated at a different distance from the presynaptic membrane, raised the possibility that each class terminated on the membrane of docked vesicles at a different distance from the presynaptic membrane. To test for this possibility, we segmented from three data sets 20 docked vesicles. The segmentations included a <3 nm-long stretch of their associated AZM macromolecules to mark the sites of connection. As shown in Figure 6A, the connection sites of each class of AZM macromolecule were localized to different domains on the vesicle surface according to distance from the presynaptic membrane. Nearest to the presynaptic membrane was a band of rib connection sites parallel to the membrane. Deep to the ribs were the connection sites of spars, and deep to the spars were the connection sites of booms (Figure 6B). Moreover, there was little overlap of the domains.

To aid in analyzing the associations of undocked vesicles with AZM macromolecules in activated axon terminals, as describe below, we determined for the 20 docked vesicles the average position of each class of connection sites relative to the presynaptic membrane and to the other classes. The average distance from the centroid of rib connections to the presynaptic membrane was ∼8 nm (7.7±3.3 nm SD), as shown previously [13]. The average distance from the centroid of the spar connections to the rib connections was ∼10 nm (10.3±4.4 nm SD). The average distance from the centroid of the boom connections to the rib connections was ∼24 nm (24.2±6.6 nm SD). The average distances between the three different classes of connection sites were distinct at a significance level of p<0.0001, as determined by ANOVA.

The connection sites of the 3–5 pins linking the presynaptic membrane to each docked vesicle were primarily on the hemisphere facing away from the main body of the AZM (Figure 7). On average, the connection sites were ∼9 nm (8.6±3.5 nm SD; n = 32 pins from 4 data sets) from the presynaptic membrane. Together with the connection sites of the ribs they formed a ring of AZM connections surrounding the vesicle membranes’ fusion domain, i.e. the area of the vesicles’ membrane that contacts the presynaptic membrane and fuses with it to release neurotransmitter.

### Associations of AZM Macromolecules with Undocked Vesicles near Vacated Docking Sites in Activated Axon Terminals

We examined by electron tomography more than 50 active zones in tissue sections from resting axon terminals for this and other studies (Jung and McMahan, unpublished results). In every case there was at least one vesicle docked on the presynaptic membrane, and each of these were linked to the AZM’s ribs, pins, spars and booms, although we did not determine for most the number and distribution of such connections as described above. At two of the active zones there was also an undocked vesicle <15 nm from the presynaptic membrane connected to the same assortment of AZM macromolecules. From electrophysiological experiments, it is evident that at neuromuscular junctions there is a relatively low frequency of vesicle fusion with the presynaptic membrane when the axon terminals are at rest as compared to when they are active [3]. Thus, the few undocked vesicles associated with ribs, spars and booms we observed at resting terminals may have been in the process of replacing former docked vesicles that had fused with the presynaptic membrane just prior to fixation.

**Figure 8:**
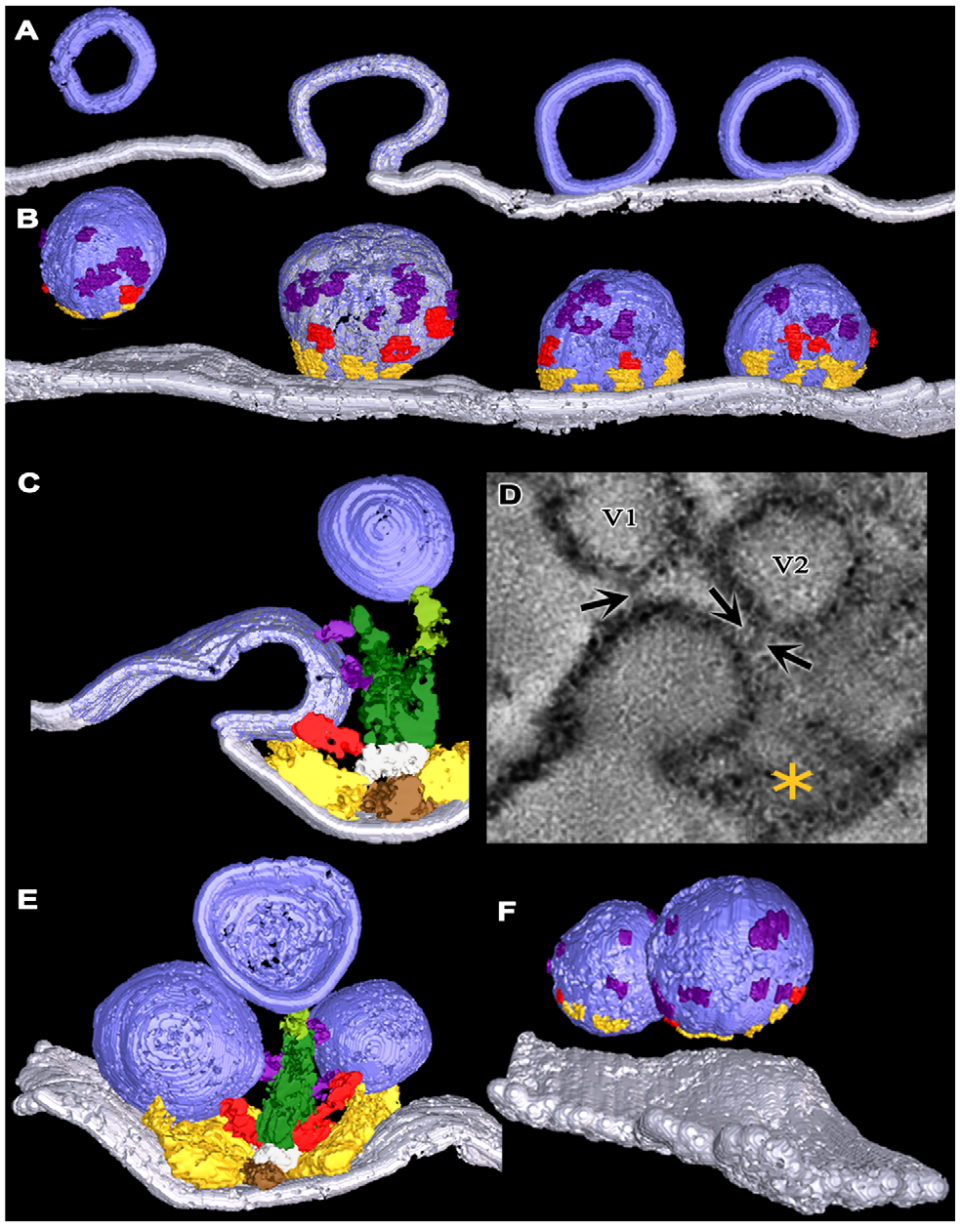
Connection of main body AZM macromolecules to synaptic vesicles at activated active zones. **A)** 10 nm thick surface model from an active zone sectioned in the median plane. It includes a row of vesicles from one side of an active zone ridge and the presynaptic membrane. Two of the vesicles are docked on the presynaptic membrane, one is fused with the membrane and one is an undocked vesicle 16 nm from a vacated docking site on the membrane. The color boundary at the interface of the fused vesicle membrane with the presynaptic membrane is arbitrary. **B)** An 80 nm thick surface model of the same vesicles in **A** that includes the vesicle surface facing the AZM. Connection sites of ribs (yellow gold), spars (red) and booms (purple) are indicated by ∼3 nm long stretches of each. **C)** A 25 nm thick surface model from an active zone sectioned in the transverse plane. A former docked vesicle that has fused with the presynaptic membrane is connected to ribs, a spar and booms. **D)** A 2.4 nm thick virtual slice, which was summed from 2 successive 1.2 nm thick virtual slices, of an activated active zone sectioned in the transverse plane. A former docked vesicle that is fused with the presynaptic membrane is connected not only to main body AZM macromolecules (asterisk), but also to non-AZM macromolecules (arrows) that link it to undocked vesicles (V1 and V2). One of the vesicles (V2) was linked to a topmast in other virtual slices. **E)** The undocked vesicle on the right of the AZM is 10 nm from a vacated docking site on the presynaptic membrane and has rib, spar, and boom connections as does the docked vesicle on the left. **F)** Undocked vesicles 10 nm and 15 nm from vacated docking sites on the presynaptic membrane with connection sites of ribs, spars and booms marked by ∼3 nm long stretches of each.

In order to observe any associations undocked vesicles might have with AZM as they replace former docked vesicles at docking sites on the presynaptic membrane, we fixed axon terminals for electron tomography while evoking synaptic activity by electrically stimulating the axons. As observed by Heuser and Reese [7], who fixed axon terminals at frog NMJ’s during such activity, the presynaptic membrane at active zones had not only sites occupied by docked synaptic vesicles but also sites where the membrane of former docked vesicles had fused with it. At some of the latter sites, the membrane of former docked vesicles had a near Ω shape. At others the vesicle membrane had flattened into the presynaptic membrane to an extent that it could not be distinguished from irregularities elsewhere in the presynaptic membrane (Figure 8).

The membrane of former docked vesicles having a near Ω shape was still connected to the same complement of booms, spars and ribs in the main body of the AZM as docked vesicles in resting terminals (Figure 8A-D). Unevenness in the presynaptic membrane near the fusion pore made it difficult to discern pins with certainty. We did not undertake to determine stages in the dissociation of the flattening vesicle membrane from AZM macromolecules as it moved laterally for retrieval because of difficulty in distinguishing flattening vesicle membrane from the general irregularities in the presynaptic membrane.

**Figure 9:**
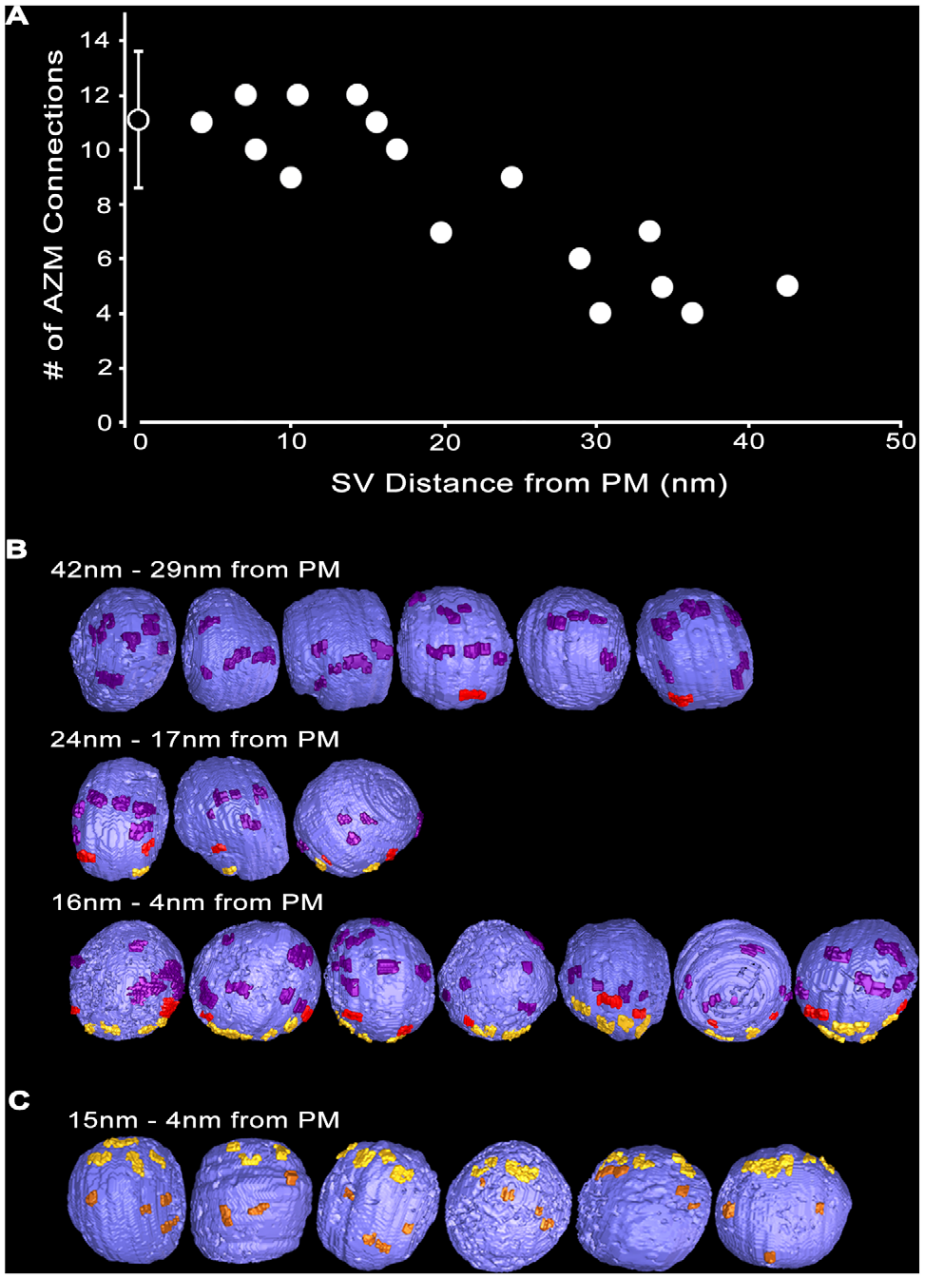
Classes of AZM macromolecules connected to undocked vesicles at various distances from vacated docking sites in activated terminals. **A)** Based on 3-D measurements in surface models from activated active zones, undocked synaptic vesicles (SV) >∼30 nm from vacated docking sites on the presynaptic membrane (PM) have relatively few connections to main body AZM macromolecules. The number of connection sites gradually increases with decreasing distance of the undocked vesicles from the presynaptic membrane to <∼15 nm, where it is similar to that for docked vesicles at resting active zones (indicated by the open symbol on the Y-axis ± SD). **B)** Viewed from the median plane of the active zone, undocked synaptic vesicles 42–29 nm from the presynaptic membrane have connection sites formed mostly by booms (purple), vesicles 24–17 nm from the presynaptic membrane have connection sites formed mostly by booms and spars (red), while vesicles 16–4 nm from the presynaptic membrane have connection sites formed by similar numbers of booms, spars and ribs (yellow gold) as on docked vesicles (compare with Figure 6A). C) Viewed in the horizontal plane, undocked vesicles 15–4 nm from the presynaptic membrane have connection sites formed by pins (copper) as well as ribs.

At vacated docking sites, i.e. sites where the fused vesicle membrane had flattened into the presynaptic membrane to the extent that the vesicle membrane could not be readily discerned, there were nearby undocked vesicles connected to the same assortment of AZM macromolecules that connected to docked vesicles in both resting and activated terminals: ribs, spars, booms and pins (Figures 8,9). For the 16 such undocked vesicles in our samples, the shortest distance between the vesicle membrane and the presynaptic membrane ranged from 42 nm to 4 nm. The total number of connections from the main body of AZM (ribs, spars and booms) on each of the vesicles was inversely proportional to the vesicle’s distance from the presynaptic membrane (Figure 9A and 9B). The vesicles that were 42–29 nm from the presynaptic membrane had, on average, ∼5 (5.2±1.2 SD) connections; those 24–17 nm from the presynaptic membrane had, on average, ∼9 (8.7±1.5 SD) connections; and those 16–4 nm from the presynaptic membrane had, on average, ∼11 (11.0±1.2 SD) connections, which was the same average number of connections found on docked vesicles in resting terminals (see above).

The number of connections formed by each of the three different classes of AZM macromolecules in the main body of the AZM with undocked vesicles was also correlated with the shortest distance of the vesicle membrane from the presynaptic membrane (Figure 9B). Undocked vesicles 42–29 nm from the presynaptic membrane had connections with, on average, ∼5 (4.8±0.8 SD) booms, which was not significantly different from the number of connections formed by booms on docked vesicles in resting terminals (5.0±2.0 SD, see above) or from the average number of boom connections on undocked vesicles closer to the docking sites on the presynaptic membrane in active terminals (5.6±1.0 SD). However, while some undocked vesicles 42–29 nm from the presynaptic membrane had connections with spars, such connections were, on average, much less frequent than those on docked vesicles at rest (0.3±0.5 SD compared to 2.2±0.5 SD; p<0.0001, t-test), and there were no connections with ribs. Undocked vesicles 24–17 nm from the presynaptic membrane had connections with, on average, ∼2 (1.7±0.6 SD) spars, which was not significantly different from the number of connections formed by spars on docked synaptic vesicles at rest (2.2±0.5 SD, see above) or from the average number of spar connections on undocked vesicles closer to the docking sites on the presynaptic membrane in active terminals (1.9±0.4 SD). However, while some undocked vesicles 24–17 nm from the presynaptic membrane had connections to ribs, such connections were at a much lower frequency per vesicle than for docked vesicles at rest (1.3±0.6 SD compared to 3.9±0.6 SD; p<0.001, t-test). Undocked vesicles 16–4 nm from the presynaptic membrane were connected to an average of ∼4 (3.6±0.5 SD) ribs, which was not significantly different from the number of connections formed by ribs on docked synaptic vesicles at rest (3.9±0.6 SD, see above). The distribution of the connection sites of the booms, spars and ribs on the surface of vesicles 16–4 nm from the presynaptic membrane was distinct for each class, as it is for docked vesicles. Using the same method for determining the average distances between ribs, spars and booms on docked vesicles (see above), for such undocked vesicles the average distance between spar and rib connections was ∼9 nm (9.3±3.6 nm SD), and the average distance between boom and rib connections was ∼26 nm (26.3±11.4 nm SD). These average distances on the undocked vesicles were not significantly different from the average distances between the connection sites of the different classes on docked vesicles (see above). Thus, within 16 nm of docking sites on the presynaptic membrane, undocked vesicles were connected to the same number and classes of macromolecules in the main body of the AZM as docked vesicles, and the relative position of the connection sites for each class was the same for both undocked and docked vesicles.

Undocked vesicles within 15 nm of docking sites on the presynaptic membrane were also connected to pins attached to the presynaptic membrane (Figure 9C). The average number of pin connections on the undocked vesicles was 3.8 ± 0.5 SD, which was not significantly different from the average of 4.1 ± 0.7 SD pin connections on docked synaptic vesicles at rest. The length of the pins varied according to the distance of the vesicles from the membrane (Figure 9C), but on average it was significantly greater (16.5 ± 6.5 nm SD; n = 21 from 6 data sets) than the length of pins connected to docked vesicles at rest (∼9 nm; Table 1) where p<0.0001 as determined by t-test.

### Filamentous non-AZM macromolecules

**Figure 10:**
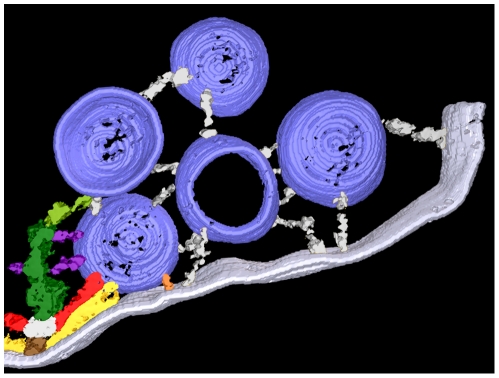
Non-AZM macromolecules connected to a docked and nearby undocked synaptic vesicles at a resting NMJ. A surface model 20 nm thick with a docked vesicle linked to AZM macromolecules colored as in previous Figures. The docked vesicle is near four undocked vesicles. Non-AZM macromolecules (pewter) link the undocked vesicles to each other and variously to the presynaptic membrane and the docked vesicle.

In addition to its connections to AZM macromolecules, each docked vesicle in resting terminals had connections to ∼10 filamentous non-AZM macromolecules. These macromolecules were up to several 10’s of nm long. They had diameters similar to those of AZM macromolecules connected to vesicles. Their connections were distributed primarily over the portion of the vesicle surface that faced away from the AZM. They linked the docked vesicles to nearby undocked vesicles, including those connected to topmasts (Figures 8D,10), or to other organelles, or they terminated blindly. We also observed such macromolecules at active zones in activated axon terminals. They were not only connected to docked vesicles but also, in similar numbers, to former docked vesicles that shared a fusion pore with the presynaptic membrane (Figure 8) and to undocked vesicles connected to AZM near vacated docking sites on the presynaptic membrane (not shown). Using the same methods that exposed the arrangement and associations of AZM macromolecules to vesicles in resting and activated terminals, we detected no obvious organization of the non-AZM macromolecules connected to the same vesicles. Moreover, we observed no difference in the number of non-AZM filaments connected to undocked vesicles at active zones in activated terminals relative to the vesicles’ distance from the presynaptic membrane.

## Discussion

**Figure 11:**
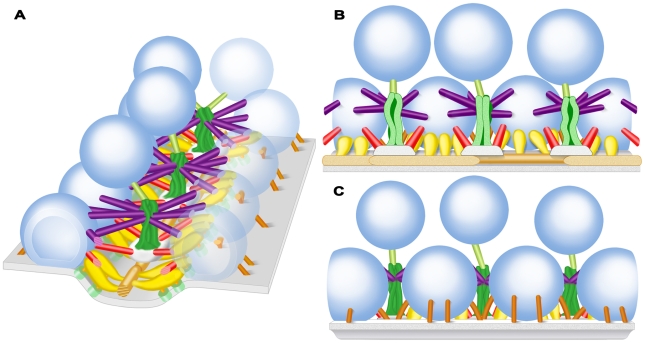
Schematized 3-D arrangement of classes of AZM macromolecules throughout the depth of the resting active zone. **A**) View includes the transverse, horizontal and median planes of the active zone (see Figure 1C). Core macromolecules include beams (brown gold), steps (grey) and masts (dark green). Macromolecules connecting core macromolecules to synaptic vesicles (dark blue) and the presynaptic membrane (pale blue) along with channels (frosted green) in the membrane, include ribs (yellow gold), pegs (orange gold), pins (copper), spars (red), booms (purple), and topmasts (light green).The presynaptic membrane and the docked vesicles in the row on the right are transparent to expose the extent of the AZM connections. **B**) View from the median plane of the active zone toward the left row of docked vesicles in **A**. **C**) View from beyond the active zone toward the left row of docked vesicles in **A**.

**Figure 12:**
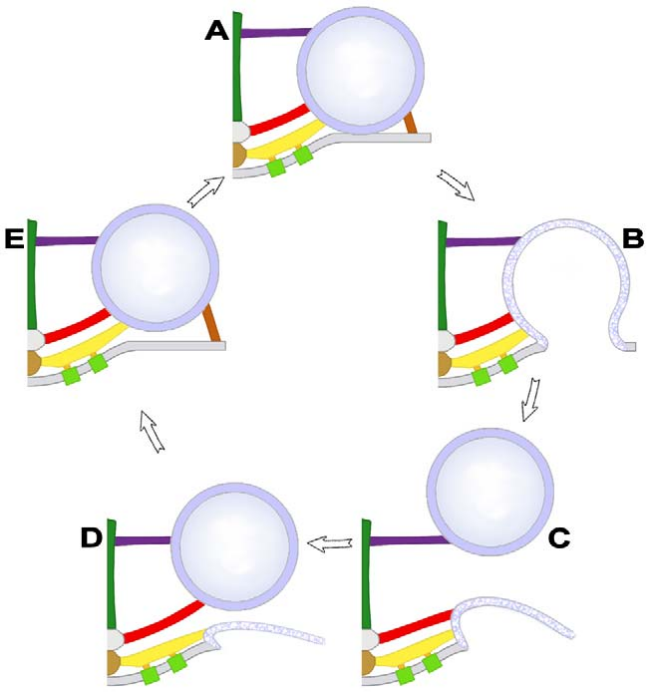
Model of the sequential association of undocked synaptic vesicles with different AZM macromolecules leading to docking. When a docked synaptic vesicle (**A**) fuses with the presynaptic membrane (**B**), booms (purple), spars (red), and ribs (yellow gold) remain attached to the vesicle membrane until it undergoes flattening into the presynaptic membrane (**C**). An undocked vesicle within 50 nm of the docking site on the presynaptic membrane first forms connections with booms that have dissociated from the fused vesicle (**C**) and then sequentially with dissociated spars (**D**) and ribs-pins (**E**) which direct it to the docking site on the presynaptic membrane (**A**). Pins are not included in **B**, **C** and **D** because we were unable to distinguish them from other presynaptic membrane linked macromolecules as the fused vesicles flattened.

We show that each docked vesicle at active zones in resting axon terminals of the frog’s NMJ is connected to multiple members of four classes of filamentous AZM macromolecules (Figure 11). The connections formed by two classes, ribs and pins, are adjacent to the presynaptic membrane and surround the domain of the vesicle that will fuse with the presynaptic membrane during synaptic transmission. The connections formed by the third class, spars, are further from the presynaptic membrane, and the connections formed by the fourth class, booms, are furthest from the membrane. At active zones in axon terminals fixed during synaptic activity, undocked vesicles within ∼50 nm vertical to sites on the presynaptic membrane formerly occupied by docked vesicles are connected to the same classes of AZM macromolecules as docked vesicles. The number of classes to which the undocked vesicles are attached is inversely related to the vesicles’ distance from the membrane. The furthest vesicles are connected primarily to the booms, nearer vesicles are connected primarily to both booms and spars, while the nearest vesicles are connected to the same assortment of classes as docked vesicles; booms, spars, ribs and pins (Figure 12). The number of connections formed by each class on undocked vesicles and the mean distance of their connection sites from each other is the same as for docked vesicles in resting axon terminals. We conclude that AZM directs undocked vesicles toward docking sites on the presynaptic membrane through a succession of specific macromolecular interactions with the vesicles, and that these same interactions persist to help hold docked vesicles in position.

Ribs, spars and booms lie in distinct layers in the main body of AZM (Figure 11). The ribs are adjacent to the presynaptic membrane, while spars are further from it, and booms are the furthest. Moreover, each of these classes arises from a different core macromolecule serially arranged vertical to the presynaptic membrane near the midline of the AZM. The ribs arise from beams, which lie next to the presynaptic membrane, while the spars arise from steps, which are connected to the deep surface of the beams, and the booms arise from the masts, which are connected to the deep surface of the steps. The pins, which are outside the AZM’s main body, arise from the presynaptic membrane and extend almost vertical to the membrane to connect to the vesicles. We include the pins as members of the AZM because their connection sites on the vesicles, together with those of ribs, are distributed around the vesicles’ fusion domain and their linkage of the vesicles to the presynaptic membrane implies that they are involved with docking of vesicles on the membrane, as are components of the main body of AZM. Thus, the identity of the different classes of AZM macromolecules to which docked vesicles are attached is based not only on the localization of their connection sites to specific domains on the vesicle surface but also on their position within the AZM and the active zone components from which they arise.

The filamentous non-AZM macromolecules we observed linking docked vesicles to undocked vesicles and to other organelles in resting terminals are similar to filamentous macromolecules that link undocked vesicles throughout axon terminals at frog NMJ’s (Xu and McMahan, unpublished) and at other synapses, e.g. [19]–[24]. Docked vesicles, former docked vesicles sharing a fusion pore with the presynaptic membrane, and undocked vesicles near vacated docking sites in activated terminals also had non-AZM macromolecules linking them to other vesicles and organelles. The disassembly and reassembly of such linkages must play a role in the regulation of vesicle movement, in general [25]. However, we did not detect, in activated terminals, differences in the number and arrangement of the connections of these macromolecules on undocked vesicles near vacated docking sites relative to the vesicles’ distance from the presynaptic membrane that could account for the movement of vesicles specifically toward the docking sites, as we did for AZM macromolecules. Because the non-AZM macromolecules were not the focus of this study, we did not include them in our surface models and schematics except for Figure 10.

There have been several electron tomography studies on synapses in the vertebrate CNS and in the nervous system of invertebrates [26], where the gross arrangement of AZM and its associated vesicles is generally more complex than at the frog’s neuromuscular junction [27]. To our knowledge only one [28], which was done on synapses of the vertebrate CNS, used data collected at a magnification sufficient to reveal structural organization of AZM at the level presented here. In that case, vesicles at and near the presynaptic membrane at active zones were shown to be associated with aggregates of macromolecules on the presynaptic membrane having a polyhedral arrangement, similar to the arrangement of macromolecules in clathrin-based coats involved in retrieval of vesicle membrane after its fusion with the presynaptic membrane. Although we observed clathrin coats beyond the active zones in resting and activated axon terminals of frog NMJ’s, we did not find such macromolecular arrangements associated with any of the more than 150 docked vesicles examined by electron tomography in this and other studies (Jung and McMahan, unpublished). The macromolecular architecture of AZM and its association with docked vesicles at the frog’s NMJ does extend to NMJ’s in mouse, even though the gross arrangement of the docked vesicles and AZM is different between the species [14]. Unlike active zones at frog NMJ’s, where each docked vesicle is positioned next to one band of AZM, at active zones of mouse NMJ’s each docked vesicle is positioned between two bands of AZM. The superficial layer of each of the two bands includes beams, ribs and pegs, which are linked to the docked vesicle and calcium channels, as at the frog’s NMJ. Thus, rib-vesicle connections have a much different distribution on the vesicle surface in mouse than in frog. Differences in the arrangement of AZM and its associated docked vesicles may influence the rate of vesicle docking and/or the probability of a docked vesicle fusing with the presynaptic membrane during synaptic transmission [14].

Based on the size of AZM macromolecules at the frog’s NMJ, it is likely that each is composed of several proteins. The apparent continuity between different classes of AZM macromolecules in our surface models raises the possibility that certain proteins contributing to one class of AZM macromolecule extend into another, while the differences in size, shape and positioning of the classes within the AZM are consistent with each class having a unique overall protein composition and function. Many, if not all, of the proteins shown by biochemistry to be involved in vesicle docking and fusion may contribute to the composition of AZM macromolecules. Such proteins include the presynaptic membrane-associated proteins syntaxin and SNAP-25 and the synaptic vesicle membrane-associated protein synaptobrevin, altogether known as SNARE proteins [29], [30]. Each SNARE protein has a domain that extends beyond the membrane with which it is associated into the cytoplasm. The cytoplasmic domains of the three proteins interact to form a complex that brings the vesicle membrane into contact with the presynaptic membrane. Synaptotagmin is another vesicle membrane-associated protein that may contribute to the AZM. Its cytoplasmic domain associates with the SNARE complex and the presynaptic membrane during docking. The binding of calcium to this domain is thought to trigger the calcium mediated fusion of docked vesicles with the presynaptic membrane during synaptic transmission [31], [32]. The cytoplasmic proteins complexin, Munc-13 and Munc-18, which are thought to regulate the interaction of the SNARE proteins [33]–[37], and Rab-3A, Rabphilin and RIM, which are thought to be involved with tethering vesicles to the docking site prior to docking [38]–[40], may also contribute to the AZM. Based on the above functional considerations it is reasonable to suggest that the cytoplasmic domains of the SNARE proteins and synaptotagmin along with the SNARE regulators, complexin, Munc-13 and Munc-18 contribute to the AZM’s ribs and pins, since these macromolecules are positioned in a way that could bring the vesicle membrane directly into contact with the presynaptic membrane during docking and influence fusion. They may, in addition, contribute to pegs (Figure 11), which are thought to also contain the cytoplasmic portion of the calcium channel [11]. Rab3A, Rabphilin and RIM might well be components of ribs, spars, booms and topmasts, because all three macromolecules appear to be involved in tethering undocked vesicles at or near the docking site prior to docking [39], [41], [42]. So-called scaffolding proteins, such as bassoon [43], piccolo [44], RIM [45], [46] and spectrin [47], which have been localized to the vicinity of active zones, may contribute the core AZM macromolecules, i.e. beams, steps, and masts. Comprehensive, quantitatively-based maps of AZM, such as the one presented here for the first time, offer the possibility of testing such hypotheses by the use of electron tomography together with immunogold labeling or protein deletion experiments, as they have been applied to the localization of proteins to structures requiring less spatial resolution than that provided by electron tomography for imaging AZM components (e.g. [22]).

Additional questions raised by this study concern how AZM macromolecules dissociate from former docked vesicles that have fused with the presynaptic membrane, and how they associate with undocked vesicles destined to dock. We demonstrate here that the fusion of docked vesicles with the presynaptic membrane occurs while the AZM macromolecules are still connected to it. Thus, the dissociation must take place as the vesicle membrane flattens into the presynaptic membrane and moves laterally for retrieval beyond the active zone. Biochemistry has provided evidence that after a vesicle fuses with the presynaptic membrane its synaptotagmin dissociates from the SNARE complex and presynaptic membrane. The proteins of the SNARE complex also disassemble so that synaptobrevin in the vesicle membrane dissociates from the syntaxin and SNAP-25 of the presynaptic membrane [48]. Accordingly, if a docked vesicle’s synaptotagmin and SNARE proteins contribute to the ribs and pins, as we suggest, a portion of the ribs and pins must, after the vesicle fuses with the presynaptic membrane, disassemble from the AZM and remain with the vesicle membrane as it flattens into the presynaptic membrane for retrieval and vesicle reformation at a distant site. Thus, undocked vesicles would carry a portion of the ribs and pins to participate in complete rib and pin reassembly in the AZM as undocked vesicles become docked. We did not undertake to determine the extent of any such disassembly and reassembly of the AZM’s ribs, pins, spars and booms during the turnover of docked vesicles. We noted, however, that the length of pins connecting undocked vesicles to the presynaptic membrane at vacated docking sites was in certain cases 3–4 times greater than the length of pins associated with docked vesicles and that the pin length was directly proportional to the undocked vesicle’s distance from the presynaptic membrane. This demonstrates that, during vesicle docking, the pins, if not all AZM macromolecules that connect to the vesicles, undergo significant structural modification.

The energy/force involved in bringing synaptic vesicles into direct contact with the presynaptic membrane is thought to be derived from the affinity that the SNARE proteins have for each other and the conformational change these proteins undergo once their interaction begins [49]. When a vesicle comes close enough to the presynaptic membrane for the cytoplasmic domain of synaptobrevin to interact with the cytoplasmic domain of syntaxin and SNAP-25, the nature of their affinity causes them to form a coil that grows incrementally to bring the vesicle membrane into contact with the presynaptic membrane. Thus, if syntaxin and SNAP-25 remain in the ribs and pins after the synaptobrevin in the membrane of former docked vesicles that have fused with the presynaptic membrane has dissociated from them, the connection of an undocked vesicle to spars at the vacated docking site might bring its synaptobrevin near enough to interact with the available syntaxin and SNAP-25. Incremental coiling within the ribs and pins could, then, cause the vesicle to move into contact with the presynaptic membrane. Such affinity driven conformational changes between vesicle and AZM proteins may also provide the energy required for the sequential association of vesicles with booms and spars during docking. However, biochemical studies indicate that the Rab3A- Rabphilin mediated tethering of undocked vesicles to components of the docking site prior to their docking is regulated by GTP-ase activity [38], [50]. Such GTP-ase regulated events may also provide energy for the sequential association of undocked vesicles with the different classes of AZM macromolecules. The results of this study raise the possibility of another source of energy in moving undocked vesicles toward the presynaptic membrane at vacated docking sites. We show that former docked vesicles sharing a fusion pore with the presynaptic membrane are linked to nearby undocked vesicles by non-AZM macromolecules. If such connections were to persist as the membrane of former docked vesicles flattens into the presynaptic membrane, the flattening could help provide the energy to bring undocked vesicles into close proximity to the booms, spars, ribs and pins, so that specific vesicle membrane components can interact with specific components of the AZM macromolecules.

Undocked vesicles lying just deep to docked vesicles and toward the midline of the AZM are linked by filamentous macromolecules to the deep end of the AZM’s masts (Figure 11). These macromolecules are grossly similar in appearance to booms and to the non-AZM macromolecules that link undocked vesicles throughout the terminal’s cytoplasm. The nearly constant presence of the macromolecules linking undocked vesicles to masts and the proximity of these undocked vesicles to docked vesicles raises the possibility that such macromolecules and/or their linkage to the masts play a specific role in active zone function. Thus, we have included them as components of the AZM, called topmasts. One function of the topmasts might be to maintain at the active zone synaptic vesicles that, because of their close proximity to the docked vesicles, preferentially replace docked vesicles after they fuse with the presynaptic membrane. Physiological experiments have shown that axon terminals have three pools of synaptic vesicles: a readily releasable pool, which has a high probability of fusing with the presynaptic membrane during synaptic transmission; a recycling pool, which replaces the vesicles in the readily releasable pool after they have fused with the presynaptic membrane; and a reserve pool, which replaces vesicles in the recycling pool as it is depleted [5]. It has long been suggested that docked vesicles correspond to the readily releasable pool [51], [52]. Our observations raise the possibility that the undocked vesicles linked by topmasts to the AZM correspond to members of the recycling pool and these vesicles move from the topmasts to booms as a stage in replacing docked vesicles that have fused with the presynaptic membrane, relying on energy from the sorts of molecular interactions that account for the sequential movement of undocked vesicles from booms to spars to ribs and pins.

Booms and spars connected to each docked vesicle and to each undocked vesicle near vacated docking sites on the presynaptic membrane typically arise from two different sets of masts and steps. The masts and steps are positioned along the AZM’s midline so the booms and spars from one mast and step approach the vesicle at a 50°–60° angle to the booms and spars from the other mast and step (Figure 11). This wide-angle trajectory of the booms and spars would have a significantly greater effect on inhibiting lateral movement of a docked vesicle, which might tend to occur, for example, when an adjacent vesicle fuses with the presynaptic membrane, than if the booms and spars approached the vesicle nearly orthogonal to the AZM’s midline, as do the ribs. For an undocked vesicle near a vacated docking site on the presynaptic membrane, its sequential connection to two sets of booms and spars having such different trajectories would favor movement of the vesicle toward the presynaptic membrane in a direction vertical to the membrane and the docking of the vesicle at precisely the same site on the membrane as the previous docked vesicle. Such limitations in the direction of movement and positioning of the undocked vesicles imposed by the vesicle’s connections to AZM might be expected if vesicle components that interact with the AZM and presynaptic membrane during docking and fusion have a specific and fixed arrangement in the vesicle membrane prior to reaching the active zone. Accordingly, the vesicle would need to approach the AZM and presynaptic membrane in a particular way for its components to interact with them properly. The vesicle’s synaptobrevin and synaptotagmin, for example, could have an arrangement in the vesicle membrane requiring the vesicle to approach the docking site at a certain degree of rotation for the synaptobrevin to interact with syntaxin and SNAP-25 in the ribs and pins and for synaptotagmin to interact both with the SNARE complex and with the presynaptic membrane. A stereotypic arrangement of vesicle proteins that interact with the spars and booms as well as ribs and pins could account for our finding that the average distances between connection sites of ribs, spars and booms on the vesicle membrane are the same for undocked vesicles near vacated docking sites on the presynaptic membrane as for docked vesicles.

## Materials and Methods

### Ethics Statement

The animal experimentation described here was approved by Stanford University’s (Protocol Number 10505) and Texas A&M University’s (AUP Number 2011–18) administrative panels on laboratory animal care (IACUC), which oversees the use of animals according to U.S. federal regulations.

### Preparation of resting neuromuscular junctions

We used *Rana pipiens* (about 5 cm nose-rump length). The paired cutaneous pectoris muscles, which are broad, flat and 1–3 muscle fibers thick, lie just beneath the skin of the frog’s chest. The muscles were prepared for electron tomography in one of three ways: 1) They were immediately exposed in terminally anesthetized (MS-222, Sigma Chemical, St Louis, Missouri) and pithed frogs. Under a dissecting microscope, 0.8% glutaraldehyde (Ted Pella, Inc., Redding, California) in Millonig’s phosphate buffer (220 mOsM total, pH 7.2) was injected beneath the muscles and dripped onto their superficial surface several times over 40 min. The muscles were removed from the frog, pinned flat in a Sylgard 184 (Dow Corning, Midland, Michigan) coated petri dish containing phosphate buffer (220 mOsM, pH 7.2) and placed on a shaker for 30 min. The muscles were then fixed and stained for 1 hr in 1% osmium tetroxide in phosphate buffer (220 mOsM total; pH 7.2), washed 1 hr in H_2_O, stained 1 hr in saturated aqueous uranyl acetate, dehydrated in increasing concentrations of ethanol and embedded flat in a wafer of Eponate 12 (Ted Pella, Inc., Redding, California) less than 1 mm thick. Regions of the muscles containing NMJs were identified in the wafers at ×400 magnification with a light microscope, and blocks were cut out and mounted for sectioning. The sections used for conventional electron microscopy shown in Figure 1 were ∼80 nm thick (Figure 1A) and ∼50 nm thick (Figure 1B) based on their interference colors. The sections used for electron tomography varied from 45 nm to 90 nm in thickness based on measurements from the reconstructed volumes. The sections were stained with uranyl acetate in methanol and with aqueous lead citrate. 2) The muscles were removed from anesthetized, pithed frogs and pinned flat in a Sylgard coated petri dish containing frog Ringer’s solution (116 mM NaCl, 20 mM KC1, 1.8 mM CaCl_2_, 10 mM sucrose, 1 mM NaH_2_PO_4_; 220 mOsM; pH 7.2) for 30 min. For fixation the Ringer’s solution was replaced by the phosphate buffered glutaraldehyde for 40 min. The muscles were then further processed as above. 3) Dr. John Heuser and Dr. Thomas Reese generously provided us with an araldite embedded block of tissue from a *Rana pipiens* cutaneous pectoris muscle that had been fixed by quick-freezing and stained with osmium tetroxide by freeze substitution (as described in [53]). Sections from this block, which contained 12 NMJs devoid of obvious ice crystal damage, were stained with uranyl acetate and lead citrate.

### Preparation of activated neuromuscular junctions

Our procedure was similar to that used by others [7] for studying by electron microscopy the behavior of synaptic vesicles at active frog NMJ’s. We used three muscles together with a 5 mm stretch of the nerve innervating each. They were pinned out in a Petri dish containing Ringer’s solution and the cut end of the nerve was drawn into a suction electrode. The activity of the preparations was tested by passing current pulses trough the suction electrode while monitoring under a dissecting microscope the muscle contractions in response to single pulses. The Ringer’s solution was then replaced by Ringer’s containing 10^–5^ g/ml (+)-Tubocurarine chloride hydrate (Sigma-Aldrich, Inc., St. Louis, Missouri) which blocked postsynaptic acetylcholine receptors and, thus, blocked muscle contractions in response to acetylcholine release, which could lead to difficulties in analyzing structural relationships in the electron tomography data. The (+)-Tubocurarine -containing Ringer’s solution was then replaced with Ringer’s solution containing 0.8% glutaraldehyde (220 mOsM total; pH 7.2), and we simultaneously began electrically stimulating the nerve with 1 ms pulses of current having an amplitude of 9–15 µA (about 10 times greater than the amount required to generate maximal muscle contraction) delivered at a frequency of 10 Hz. The stimulation continued for 2 min. We had previously observed under a dissecting microscope that in stimulated nerve-muscle preparations not exposed to (+)-Tubocurarine, contractions of all muscle fibers had ceased after 2 min in the fixative, which indicated that all of the NMJ’s were fixed at that time. After stimulation ceased, the muscles remained in fixative for 40 min. They were then further processed for electron tomography according to the second method used for preparing resting NMJ’s described above.

### Data collection

Data sets were collected using one of two electron microscopes designed for automatic data acquisition: 1) a Phillips Tecnai T20 electron microscope (FEI Company Hillsboro, Oregon) equipped with a 1024×1024 CCD (Gatan, Inc., Pleasanton, California) in the laboratory of Dr. David Agard at the University of California, San Francisco; and 2) an FEI TF30 Polara electron microscope (FEI Company Hillsboro, Oregon) equipped with a 2048×2048 Tietz TemCam-F224HD CCD (Tietz Video and Imaging Processing Systems GmbH, Gauting, Germany) in our own laboratory at Stanford University. The stage on each microscope was cooled to liquid nitrogen temperature to reduce specimen shrinkage. Most data sets consisted of images taken at 1-degree tilt intervals to ±60 or ±70 degrees along a single tilt axis. One was taken at 2-degree tilt intervals to ±60 degree, while others were taken at 1-degree intervals to ±60 along each of two orthogonal tilt axes. Reconstructions from the dual axis data sets have less noise than those from single axis data sets [54], [55]. However, both types of data sets yielded qualitatively similar structural models, and measurements from both types rose to statistical significance.

For generating reconstructions, the images were first aligned automatically using 5 or 10 nm gold colloid (British Biocell International, Cardiff, U.K.) deposited on one or both sides of the sections before data collection as fiducial markers. The average alignment error of data sets in this study was less than 1 pixel (range, 0.5 to 1.6) root mean square. The reconstructions were made by a weighted back-projection method. Both the alignment and reconstruction algorithms are in the unified software package for electron tomography, EM3D (em3d.stanford.edu) [13], [56]. The spatial resolution was 2–3 nm for high contrast structures such as the cytoplasmic and extracellular layers of the plasma membrane [56].

None of the vesicles in our data sets were perfectly spherical. However, in most data sets there was no significant difference in the average dimensions of vesicles measured in all three axes. In data sets that did exhibit compression in the z axis of the section, probably due to thinning of the plastic caused by prolonged exposure to the electron beam [57], the z-axis diameters of the vesicles were 80.5 ± 4.8% (mean ± SD, n = 15 vesicles) of the diameters measured in the x-y axis of the section. Thus, a ‘stretch’ factor [58] of 1.2 was applied to the z-axis of the volumes reconstructed from these data sets.

### Virtual slices, segmentation and rendering surface models

Virtual slices through the reconstructed tissue sections were 1 voxel thick. Depending on the magnification of the images in a data set, the virtual slice thickness represented 0.52 nm to 1.2 nm of the tissue section’s thickness. When necessary, the angular orientation of the slice plane was adjusted to maximize contrast boundary discrimination of the structures under study.

Structures were segmented from the reconstructions by using a combination of manual and semi-automatic methods in EM3D to define individual volumes-of-interest (VOIs; [13]). For the presynaptic membrane and synaptic vesicles, which were heavily stained and had a simple geometry, a semi-automatic scheme was used and manually adjusted as necessary. For structures that had a complex geometry and light to moderate stain, VOIs were defined by manually marking a closed path on the series of slices in which they were included. The VOIs for both the semi-automatic and manual methods were slightly larger than the structures that they enclosed to allow accurate and complete isodensity-surface calculations for the surface models.

We used EM3D to render a surface model from each VOI. The rendering was done using a gray scale value that minimized the mean spatial uncertainty averaged across the whole area of the model. Surface models generated in this way had a spatial resolution equal to the resolution of the reconstructed volumes [13].

### Dimensions and Angles of Approach

The dimensions of the AZM macromolecules displayed in Table 1 were obtained as follows. To rule out any effects from missing wedge artifact that might result from the tilt-angle limitation in data collection, we used reconstructions from several tissue sections, each from one of the three cardinal planes of the active zone: horizontal, transverse and median (Figure 1C). For determining lengths, the reconstructed volume was rotated so the full length of each macromolecule was included in a single virtual slice made along the macromolecule’s midline, and the longitudinal extent was measured using the “measurement” tool in EM3D [13]. The diameters of steps and masts varied along their length. For those in Table 1 a virtual slice transverse to the long axis of each structure was made midway along its length, and the diameters were measured on the cross-section using the “measurement” tool in EM3D. For convenience as well as accuracy, we determined the average diameter of beams, ribs, spars, booms, topmasts and pins, which were more uniform in diameter than the steps and masts, in surface models using the “thickness” tool in EM3D [13].

To determine the angle the ribs, spars and booms approached docked vesicles, the reconstructed volume of a tissue section was rotated so that the beam(s) could be viewed in the horizontal plane of the active zone. All of the AZM macromolecules and synaptic vesicles were then projected onto a 2-D plane [14] and the projection was rotated so that the long axis of the beam(s) was aligned with and centered on a virtual reference line running horizontal to the field of view (see dashed line in Figure 5A). An ‘adjacent’ line was then drawn at a right-angle from the reference line to the centroid of the connection site of a rib, spar or boom on a vesicle. A hypotenuse was drawn along the long axis of each AZM macromolecule from the centroid to the reference line. The angle between the hypotenuse and the adjacent line, which was calculated by standard trigonometry, represented the angle of approach of ribs, spars and booms to vesicles.

### Distribution of connection sites on docked vesicles

The AZM connection sites on each synaptic vesicle were first mapped onto a unit sphere based on the spatial coordinates of the centroids of connection sites and the diameter of the individual vesicle to normalize the variability of vesicle diameters. All of the vesicles were then rotated so their connection sites faced the same direction, which provided a rough alignment. For fine-alignment of the vesicles to the position of the rib connections (in *x, y, z* coordinates), the degree of overlap of rib connections for all vesicles was maximized based on the following equation:
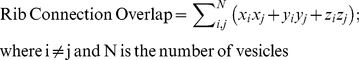



To calculate the rotation angle with maximized values for *Rib Connection Overlap*, we used Euler’s rotation theorem [59]. All vesicles were then rotated to their calculated value, and all AZM connections were plotted onto the common unit sphere shown in Figure 6B.

The direct distance from the centroid of each connection site to the closest point on the presynaptic membrane was measured using the proximity tool in EM3D and then normalized to the average position of the rib connection sites per individual vesicle. These normalized measurements were plotted in Figure 6C.

### Figure layouts

Figure layouts were prepared using Adobe Photoshop CS3 (Adobe Systems, San Jose, CA). The grey-scale levels and curves for Figure 1 were adjusted slightly in Photoshop in order to optimize the fidelity of the electron micrograph images for publication and reproduction.

The RGB color values for the surface models are as follows: presynaptic membrane (200,200,225); synaptic vesicles (125,125,255); ribs (255,197,31); beams (125,75,25); pegs (255,175,0); pins (200,100,25); steps (240,240,240); spars (255,0,0); masts (0,100,0); booms (100,0,150); topmasts (100,150,0); non-AZM macromolecules (200,200,200).
